# Knockdown of Rab9 Recovers Defective Morphological Differentiation Induced by Chemical ER Stress Inducer or PMD-Associated PLP1 Mutant Protein in FBD-102b Cells

**DOI:** 10.3390/pathophysiology31030032

**Published:** 2024-08-26

**Authors:** Nana Fukushima, Yuki Miyamoto, Junji Yamauchi

**Affiliations:** 1 Laboratory of Molecular Neuroscience and Neurology, Tokyo University of Pharmacy and Life Sciences, Tokyo 192-0392, Japan; s21802@toyaku.ac.jp (N.F.); miyamoto-y@ncchd.go.jp (Y.M.); 2 Laboratory of Molecular Pharmacology, National Research Institute for Child Health and Development, Tokyo 157-8535, Japan; 3 Diabetic Neuropathy Project, Tokyo Metropolitan Institute of Medical Science, Tokyo 156-8506, Japan

**Keywords:** Rab9, oligodendrocyte, morphogenesis, ER stress, hypomyelinating leukodystrophy

## Abstract

Small GTP-binding proteins of the Rab family regulate intracellular vesicle trafficking across many aspects of the transport system. Among these, Rab9 is recognized for its role in controlling the transport system not only around the trans-Golgi network but also around the late endosome. However, the specific functions across different cell types and tissues remain unclear. Here, for the first time, we report that Rab9 negatively regulates morphological changes in the FBD-102b cell line, an oligodendroglial precursor cell line undergoing morphological differentiation. The knockdown of Rab9 led to an increase in cell shape alterations characterized by widespread membrane extensions. These changes were accompanied by increased expression levels of oligodendroglial cell differentiation and myelination marker proteins. Notably, the knockdown of Rab9 was capable of recovering defective cell morphological changes induced by tunicamycin, an inducer of endoplasmic reticulum (ER) stress, which is one of the major causes of oligodendroglial cell diseases such as Pelizaeus–Merzbacher disease (PMD, currently known as hypomyelinating leukodystrophy type 1 [HLD1]). In addition, Rab9 knockdown recovered levels of ER stress marker proteins and differentiation markers. Similar results were obtained in the cases of dithiothreitol (DTT), another chemical ER stress inducer, as well as HLD1-associated proteolipid protein 1 (PLP1) mutant protein. These results indicate a unique role for Rab9 in oligodendroglial cell morphological changes, suggesting its potential as a therapeutic target for mitigating diseases such as HLD1 at the molecular and cellular levels.

## 1. Introduction

The central nervous system (CNS) is composed of a variety of tissues and cells, including neuronal and glial cells [1,2,3,4]. Oligodendrocytes (also called oligodendroglial cells) form myelin sheaths by wrapping neuronal axons with their differentiated plasma membranes [5,6,7,8]. These myelin sheaths play crucial roles in the efficient transmission of nerve impulses and the protection of axons. However, when myelin sheaths are not properly formed or are damaged, inflammatory [9,10] or genetic [11,12,13,14,15,16] neurodegradative diseases can occur.

Rab proteins, small GTP/GDP-binding proteins belonging to the Ras superfamily, are known to control almost all intracellular vesicle trafficking [17,18,19,20]. These proteins exist in two forms: the active GTP-bound form and the inactive GDP-bound form [17,18,19,20]. They are regulated by GTPase-activating proteins (GAPs), which generate the GDP-bound form, and GDP/GTP exchange factors (GEFs), which generate the GTP-bound form [17,18,19,20]. Each Rab protein localizes to specific cellular vesicles, membranes, or tissues and plays a role in controlling vesicular budding, trafficking, and fusion, impacting various cell physiologies in both health and disease states [21,22]. Among Rab proteins, Rab9 is broadly localized in trans-Golgi networks, late endosomes, and other organelles [23,24,25]. It controls the recycling of mannose-6-phosphate receptor protein, a typical intracellular recycling vesicle marker around trans-Golgi networks [23,24,25]. Rab9 also contributes to the sorting and transport of proteins in lysosomes [26,27,28], facilitating the regulation of lysosome biogenesis and acting as a bridge in the construction of the autophagy system [26,27,28].

The endoplasmic reticulum (ER) plays a central role not only in intracellular calcium storage but also in protein quality control through the folding of secretory and membrane proteins [29,30,31,32]. When cells are exposed to external and/or internal stresses, they generate a set of reactions and proteins known as the unfolded protein response (UPR) [29,30,31,32]. Under prolonged or excessive stress conditions, these unfolded proteins accumulate, resulting in impaired cell growth and differentiation. Consequently, ER stress can contribute to diseases affecting various tissues and organs. Among them, CNS oligodendroglial cell-related diseases typically include the *proteolipid protein 1* (*plp1*) gene-related hypomyelinating leukodystrophy 1 [HLD1] (also called Pelizaeus–Merzbacher disease [PMD]) and other HLDs [33,34,35,36]. HLD1 and certain HLDs manifest as recessive diseases in which oligodendroglial cell-derived differentiated myelin sheath membranes are improperly formed [11,12,13,14,15,16].

Rab9 is divided into two different subtype molecules: Rab9A and Rab9B. Since Rab9A is typically regarded as a general Rab9, we consider Rab9A as synonymous with the Rab9 molecule [23,24,25,26,27,28]. Among the many oligodendroglial cell Rab proteins [37,38,39,40], we focused on Rab9 in this study. We previously reported that Rab7A and Rab7B are involved in the antagonistic regulation of morphological differentiation in the FBD-102b cell line, a model for oligodendroglial cell differentiation [41,42]. Given that Rab7 subfamily members include Rab9 [23,24,25,26,27,28], we examined whether Rab9 also affects morphological differentiation. Here, we investigated the effects of Rab9 using small interfering (si)RNA on morphological differentiation. We found that the knockdown of Rab9 resulted in decreased morphological changes with oligodendroglial cell-like widespread membranes. This observation prompted us to test the possibility that its knockdown could recover the inhibition of morphological changes due to chemically induced ER stress, a condition similar to the cause of HLD1 and other neurodegradative diseases [43,44,45]. In addition, we tested the effects of Rab9 knockdown on cellular phenotypes induced by ER stress-related HLD1-associated mutant protein [33,34,35,36]. These molecular and cellular studies suggest that Rab9 could serve as a potential drug target for oligodendroglial cell diseases induced by ER stress.

## 2. Materials and Methods

### 2.1. Materials

Key materials, including antibodies, chemicals, and nucleotides, are listed in Table 1.

### 2.2. Cell Culture and Differentiation

(1) The FBD-102b cell line (kindly provided by Dr. Yasuhiro Tomo-oka [Riken, Saitama, Japan/Tokyo University of Science, Chiba, Japan]), a mouse oligodendroglial precursor cell line, was cultured in Dulbecco’s modified Eagle medium (DMEM)/Ham’s F-12 nutrient mixed medium (Nacalai Tesque, Kyoto, Japan; Fujifilm, Tokyo, Japan) containing 10% heat-inactivated fetal bovine serum (FBS) and PenStrep mixed antibiotics (Thermo Fisher Scientific, Waltham, MA, USA).
(2) The cells were seeded at 2 × 10^6^ per 10 cm diameter of Nunc cell and tissue culture dishes (Thermo Fisher Scientific, Waltham, MA, USA) and grown until 8 × 10^6^ per 10 cm diameter of culture dishes.
(3) The dishes were cultured in 5% carbon dioxide at 37 °C until 20 passages.
(4) To induce differentiation, the cells were plated at 4 × 10^6^ per 10 cm diameter polylysine (Nacalai Tesque)-coated cell culture dishes in culture medium containing 1% FBS and maintained in 5% carbon dioxide at 37 °C.
(5) The cells were allowed to be differentiated for several days.
(6) The cells displaying secondary branches from primary ones or exhibiting myelin membrane-like widespread membranes (cells large enough to contain a circle with a diameter exceeding 0.03 mm) were considered to have differentiated phenotypes [39].
(7) Cell morphologies were captured using a microscopic system equipped with i-NTER LENS (Micronet, Saitama, Japan) and i-NTER SHOT ver.2 (Micronet).
(8) The images in the figures are representative of multiple captures and were analyzed with Image J software (ver. Java 8, https://imagej.nih.gov/ on 1 July 2024).

### 2.3. Reverse Transcription and Polymerase Chain Reaction (RT-PCR) and Routine PCR

(1) Total cellular RNA was prepared from cells grown on a 10 cm diameter culture dish using Isogen (Nippon Gene, Tokyo, Japan) according to the manufacturer’s instructions.
(2) Single-strand cDNAs were generated from their RNAs (1 mg of RNAs per one reaction) using the PrimeScript RT Master Mix kit (Takara Bio, Kyoto, Japan) in accordance with the manufacturer’s instructions.
(3) PCR amplification was performed using 1:40 of the RT product with Gflex DNA polymerase (Takara Bio) for 35 cycles.
(4) Each cycle consisted of a denaturation reaction at 98 °C (0.2 min), an annealing reaction at 56 to 65 °C (0.25 min), depending on the annealing temperature, and an extension reaction at 68 °C (0.5 min).
(5) The resultant PCR products were loaded onto premade agarose gels (Nacalai Tesque; Fujifilm).

### 2.4. siRNA and/or Plasmid Transfection

(1) The cells were transfected with the respective siRNAs using the ScreenFect siRNA transfection kit (Fujifilm) in accordance with the manufacturer’s instructions. Alternatively, the cells were cotransfected with the respective siRNAs and plasmids using the ScreenFect A transfection kit (Fujifilm).
(2) The medium was replaced with fresh medium 4 h after transfection.
(3) The cells were treated with chemicals or their vehicle controls at 24 h after transfection.
(4) The cells were generally used for biochemical experiments more than 48 h after transfection.

### 2.5. Cell Lysis and Polyacrylamide Gel Electrophoresis

(1) The cells were lysed in cell and tissue extraction buffer (50 mM HEPES-NaOH, pH 7.5, 150 mM NaCl, 3 mM MgCl_2_, 1 mM dithiothreitol, 1 mM phenylmethane sulfonylfluoride, 1 μg/mL leupeptin, 1 mM EDTA, 1 mM Na_3_VO_4_, and 10 mM NaF) with a mild detergent (0.5% NP-40) [39]. Generally, 0.1 mL of extraction buffer was used per 1 × 10^6^ cells.
(2) For denaturing conditions, cell lysates were mixed with premade sample buffer (Fujifilm) in accordance with the manufacturer’s instructions.
(3) Denatured samples (0.025 mg of proteins per lane) were separated on premade sodium dodecyl sulfate-polyacrylamide gel (Nacalai Tesque).

### 2.6. Blotting and Detection of Immunoreactive Bands

(1) Electrophoretically separated proteins were transferred to a polyvinylidene fluoride membrane (Fujifilm).
(2) The membrane was blocked using the Skim Blocker kit (Fujifilm).
(3) The blocked membrane was immunoblotted with primary antibodies.
(4) This was followed by incubation with peroxidase enzyme-conjugated secondary antibodies.
(5) The bands were chemically reacted with the Chemi-Lumi One Super or Ultra kit (Nacalai Tesque).
(6) Peroxidase-reactive bands were detected on X-ray film (Fujifilm) at the same exposure time (within 0.5 h) for each sample being compared.
(7) The reactive bands on the film were captured using the CanoScan LiDE 400 (Canon, Tokyo, Japan) and CanoScan LiDE 400 Scanner Driver Ver.1.01.
(8) Multiple sets of experiments were conducted for immunoblotting studies, and the quantification of immunoreactive bands was performed using Image J software (ver. Java 8), with the immunoreactive band of another sample set as the 100% reference.

### 2.7. Statistical Analyses

(1) For all analyses, the investigator was blinded to the sample conditions. The values are expressed as means ± standard deviation (SD) from separate experiments.
(2) Intergroup comparisons were performed using the unpaired *t*-test with Student’s correction or Welch’s correction, conducted with Excel software (ver. 2024, Microsoft, Redmond, WA, USA).
(3) Differences were considered significant at *p* < 0.05.

### 2.8. Ethics Statement

Techniques involving genetically modified cells and related procedures were performed in accordance with a protocol approved by the Tokyo University of Pharmacy and Life Sciences Gene and Animal Care Committee (Approval Nos. LS28-20 and LSR3-011).

## 3. Results

### 3.1. Rab9 Negatively Regulates Cell Morphogenesis

Rab9 is composed of two different homologues, Rab9A and Rab9B. We thus treat Rab9A as a Rab9 molecule, given its recognition as a general Rab9 [23,24,25,26,27,28]. We knocked down Rab9 using its specific siRNA in FBD-102b cells (Figure S1).

**Figure 1:**
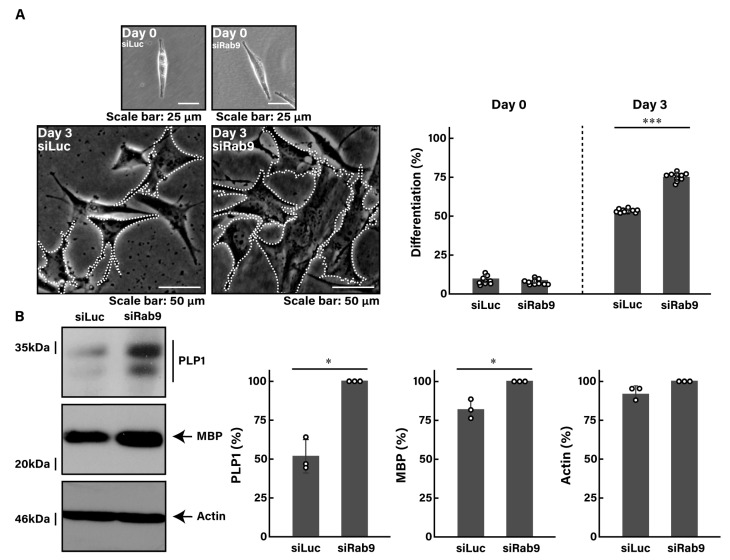
Knockdown of Rab9 promotes cell morphological changes. (**A**) FBD-102b cells were transfected with control luciferase siRNA (siLuc) or Rab9 siRNA (siRab9). Following the induction of differentiation, cell morphologies were photographed and cells with differentiated oligodendroglial cell-like widespread membranes were statistically depicted at day 0 or 3 (*** *p* < 0.001; *n* = 10 fields). Typical cell morphologies with differentiated oligodendroglial cell-like widespread membranes were surrounded by white dotted lines. (**B**) Cell lysates at day 3 following the induction of differentiation were immunoblotted with the respective antibodies against PLP1, MBP, and internal control actin and statistically depicted as a percentage comparison (* *p* < 0.05; *n* = 3 blots).

Following the induction of differentiation, control luciferase-knocked down cells typically exhibited approximately 50% of differentiated phenotypes with mature oligodendrocyte-like widespread membranes at 3 days. In contrast, Rab9-knocked down cells showed a notable increase in phenotypes with widespread membranes, reaching approximately 75% (Figure 1A). These results were consistent with increased expression levels of oligodendroglial cell differentiation and myelination marker proteins, including proteolipid protein 1 (PLP1) and myelin basic protein (MBP) (Figure 1B). The expression levels of the internal control marker protein actin remained comparable between control- and Rab9-knocked down cells.

These results suggest that Rab9 knockdown can lead to promoting morphological differentiation.

### 3.2. Knockdown of Rab9 Leads to Recovering Tunicamycin-Induced Cell Phenotypes

**Figure 2:**
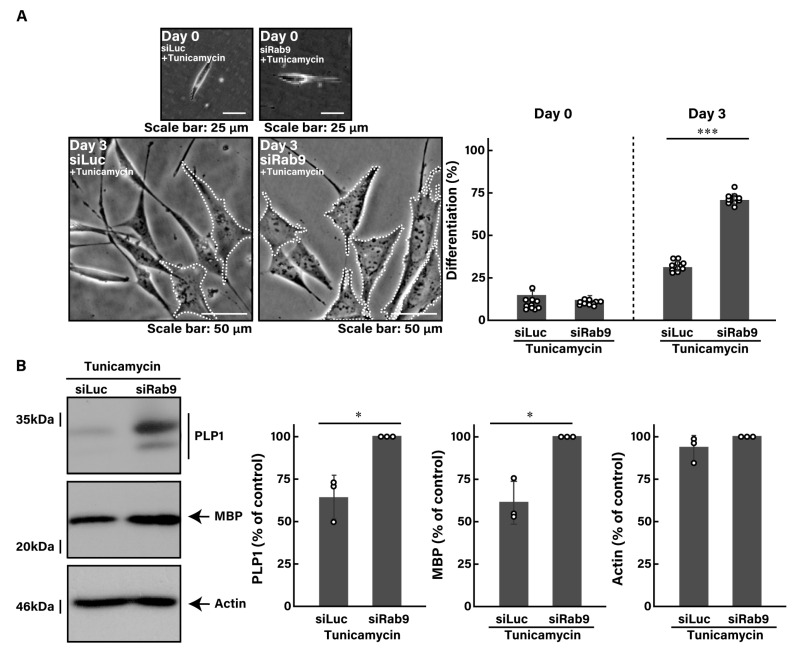
Knockdown of Rab9 recovers phenotypes induced by tunicamycin. (**A**) FBD-102b cells were transfected with luciferase siRNA (siLuc) or Rab9 siRNA (siRab9). Following the induction of differentiation in the presence of 100 ng/mL of tunicamycin, cell morphologies were photographed and cells with differentiated oligodendroglial cell-like widespread membranes were statistically depicted at day 0 or 3 (*** *p* < 0.001; *n* = 10 fields). Typical cell morphologies with differentiated oligodendroglial cell-like widespread membranes were surrounded by white dotted lines. (**B**) Cell lysates at day 3 were immunoblotted with the respective antibodies against PLP1, MBP, and actin and statistically depicted as a percentage comparison (* *p* < 0.05; *n* = 3 blots).

To investigate whether Rab9 knockdown can recover disease-like phenotypes caused by ER stress in oligodendroglial cells, we first treated FBD-102b cells with tunicamycin, an inducer of UPR stress signaling in the ER [43,44,45]. Treatment with tunicamycin resulted in a reduction in morphological differentiation by approximately 30% (Figure 2A, compared to Figure 1A).

Tunicamycin not only decreased the expression levels of MBP, an oligodendroglial cell differentiation marker, but also increased the expression levels of heat shock protein family A member 5 (HSPA5, also called immunoglobulin heavy chain-binding protein [BiP] or 78 kDa glucose-regulated protein [GRP78]) and CCAAT/enhancer-binding protein homologous protein (CHOP, also called C/EBP6 or DNA damage-inducible transcript 3 [DDIT3]), the major markers of ER stress signaling (Figure S2). In addition, it elevated the phosphorylation levels of eukaryotic translation initiation factor 2A (eIF2a), another output of ER stress signaling (Figure S2).

**Figure 3:**
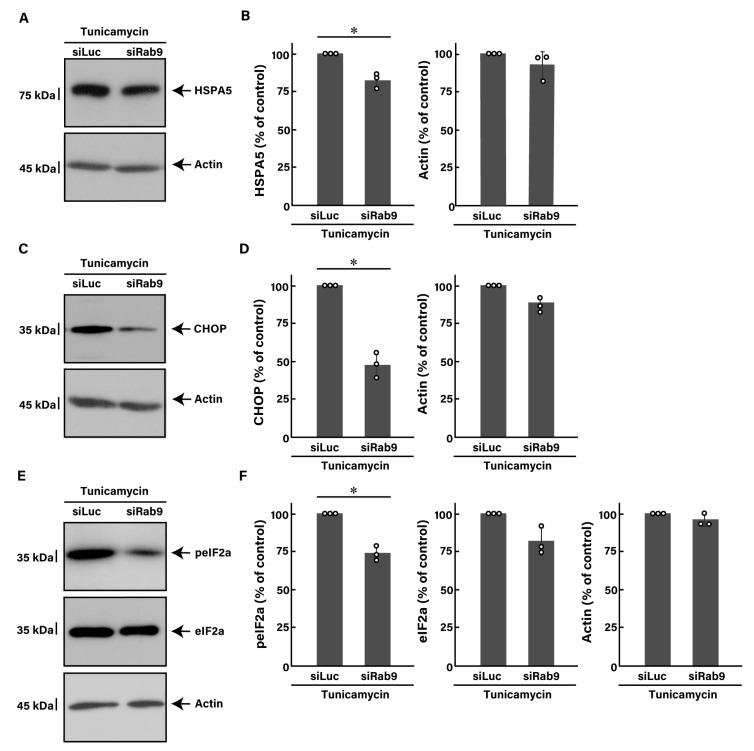
Knockdown of Rab9 decreases tunicamycin-induced ER stress signaling. (**A**,**B**) FBD-102b cells were transfected with luciferase siRNA (siLuc) or Rab9 siRNA (siRab9). Following the induction of differentiation in the presence of 100 ng/mL of tunicamycin, cell lysates at day 3 were immunoblotted with the respective antibodies against HSPA5 and actin and statistically depicted as a percentage comparison (* *p* < 0.05; *n* = 3 blots). (**C**,**D**) Following the induction of differentiation in the presence of 100 ng/mL of tunicamycin, cell lysates were immunoblotted with the respective antibodies against CHOP and actin and statistically depicted as a percentage comparison (* *p* < 0.05; *n* = 3 blots). (**E**,**F**) Following the induction of differentiation in the presence of 100 ng/mL of tunicamycin, cell lysates were immunoblotted with the respective antibodies against phosphorylated eIF2a (peIF2a), eIF2a, and actin and statistically depicted as a percentage comparison (* *p* < 0.05; *n* = 3 blots).

Next, we investigated whether Rab9 knockdown has the ability to recover tunicamycin-induced decreases in morphological differentiation. The knockdown of Rab9 recovered phenotypes with widespread membranes by approximately 50 to 60% (Figure 2A), consistent with increased expression levels of PLP1 and MBP (Figure 2B). In contrast, the expression levels of actin were comparable between control- and Rab9-knocked down cells. In addition, Rab9 knockdown recovered the expression levels of both HSPA5 (Figure 3A,B) and CHOP (Figure 3C,D), as well as the phosphorylation levels of eIF2a (Figure 3E,F), illustrating that Rab9 knockdown has protective effects against tunicamycin-induced phenotypes.

### 3.3. Knockdown of Rab9 Leads to Recovering Dithiothreitol (DTT)-Induced Cell Phenotypes

We investigated whether Rab9 knockdown can recover cellular phenotypes induced by ER stress under another pharmacological stress condition. We thus examined whether treatment with dithiothreitol (DTT), another inducer of stress signaling and UPR in the ER [43,44,45], stimulates ER stress signaling. Indeed, treatment with DTT increased the expression levels of both HSPA5 and CHOP, along with the phosphorylation levels of eIF2a, while decreasing the expression levels of MBP marker proteins (Figure S3).

**Figure 4:**
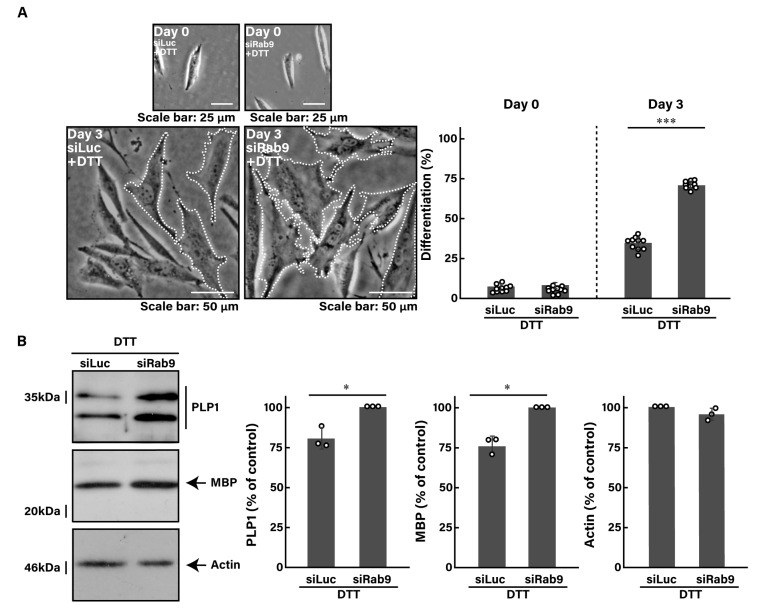
Knockdown of Rab9 recovers phenotypes induced by DTT. (**A**) FBD-102b cells were transfected with luciferase siRNA (siLuc) or Rab9 siRNA (siRab9). Following the induction of differentiation in the presence of 1 mM DTT, cell morphologies were photographed and cells with differentiated oligodendroglial cell-like widespread membranes were statistically depicted at day 0 or 3 (*** *p* < 0.001; *n* = 10 fields). Typical cell morphologies with differentiated oligodendroglial cell-like widespread membranes were surrounded by white dotted lines. (**B**) Cell lysates at day 3 were immunoblotted with the respective antibodies against PLP1, MBP, and actin and statistically depicted as a percentage comparison (* *p* < 0.05; *n* = 3 blots).

**Figure 5:**
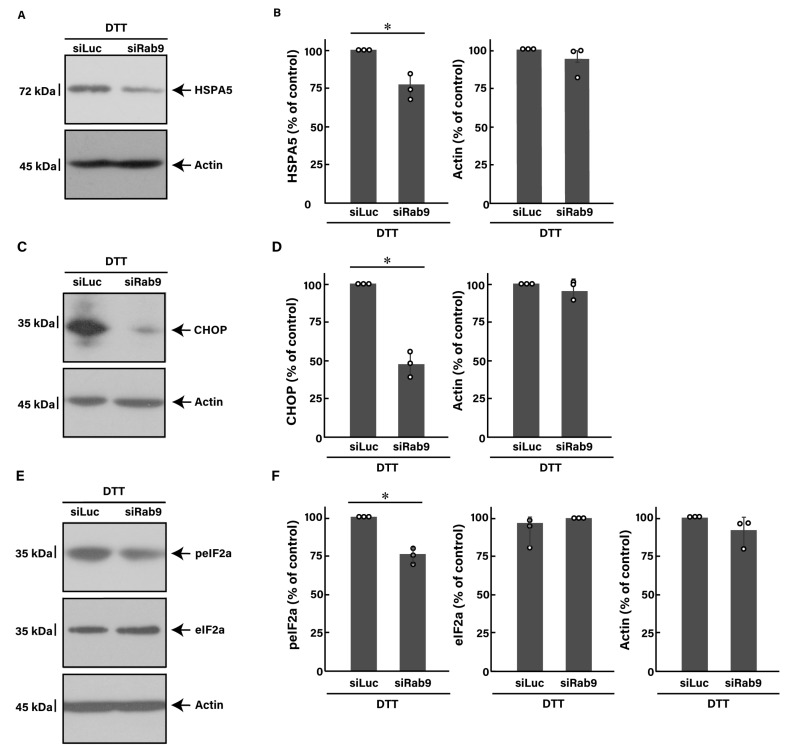
Knockdown of Rab9 decreases DTT-induced ER stress signaling. (**A**,**B**) FBD-102b cells were transfected with luciferase siRNA (siLuc) or Rab9 siRNA (siRab9). Following the induction of differentiation in the presence of 1 mM DTT, cell lysates at day 3 were immunoblotted with the respective antibodies against HSPA5 and actin and statistically depicted as a percentage comparison (* *p* < 0.05; *n* = 3 blots). (**C**,**D**) Following the induction of differentiation in the presence of 1 mM DTT, cell lysates were immunoblotted with the respective antibodies against CHOP and actin and statistically depicted as a percentage comparison (* *p* < 0.05; *n* = 3 blots). (**E**,**F**) Following the induction of differentiation in the presence of 1 mM DTT, cell lysates were immunoblotted with the respective antibodies against phosphorylated eIF2a (peIF2a), eIF2a, and actin and statistically depicted as a percentage comparison (* *p* < 0.05; *n* = 3 blots).

Similar to the finding with tunicamycin, we investigated whether the knockdown of Rab9 can recover the DTT-induced decrease in morphological differentiation. The knockdown of Rab9 recovered phenotypes with widespread membranes by 50 to 60% (Figure 4A), consistent with the increased expression levels of PLP1 and MBP (Figure 4B). In addition, Rab9 knockdown recovered both the expression levels of HSPA5 (Figure 5A,B) and CHOP (Figure 5C,D), as well as the phosphorylation levels of eIF2a (Figure 5E,F). Collectively, Rab9 knockdown has protective effects against DTT-induced phenotypes.

### 3.4. Knockdown of Rab9 Leads to Recovering Cell Phenotypes Induced by PLP1 with the A243V Mutation

To further confirm whether Rab9 knockdown can recover cellular phenotypes induced by a known ER stress inducer in oligodendroglial cells, we transfected cells with PLP1 containing the A243V mutation, which is well known to be associated with ER stress-related HLD1 [11,12,13,14,15,16]. Transfection with PLP1 A243V decreased morphological differentiation, as indicated by reduced expression levels of GSTpi, oligodendroglial cell differentiation markers other than markers such as PLP1, and increased levels of HSPA5 (Figure S4).

Next, we explored whether Rab9 knockdown could recover the decrease in morphological differentiation induced by PLP1 with the A243V mutation. The knockdown not only restored phenotypes with widespread membranes and increased GSTpi expression levels but also reduced HSPA5 expression levels (Figure S5), suggesting that Rab9 knockdown has protective effects against the phenotypes induced by PLP1 with the A243V mutation.

### 3.5. Effects of ER Stress Inducers and Rab9 Knockdown on Akt Signaling

We examined how Rab9 knockdown affects signaling through Akt kinase, a major signal transducer in oligodendroglial differentiation [3,4,5,6,7,8], under ER stress conditions. Since Akt phosphorylation is related to its kinase activity and oligodendroglial differentiation [14,15], we investigated whether treatment with tunicamycin, DTT, or transfection with the plasmid encoding PLP1 with the A243V mutation decreases Akt phosphorylation levels. All of these treatments resulted in reduced Akt phosphorylation; however, Rab9 knockdown restored these levels (Figure S6). This suggests a potential parallel relationship between Rab9 knockdown and differentiation-associated signaling.

## 4. Discussion

Rab9 actually exhibits wide distribution in vesicles around the trans-Golgi network and plays multiple roles in intracellular vesicle transport. Rab9 is also present around late endosomes, facilitating the sorting and transporting proteins to the lysosome [23,24,25,26,27,28]. It is believed to act as a bridge in the construction of the autophagy system, aiding in the formation of autolysosomes by fusing lysosomes with autophagosomes [26,27,28].

Despite the well-known essential functions of Rab9 in the intracellular vesicle transport system, it remains unclear whether Rab9 is involved in forming specific cell phenotypes in the respective cell types or whether it serves as a simple housekeeping gene product. If Rab9 participates in certain cellular phenotypes, the question arises as to what role it plays in the respective cell types. Studies using knockout cells and mice have illustrated that some Rab proteins are specifically involved in the regulation of cell morphogenesis in certain cell types [21,22]. For example, the knockout of Rab7, a Rab family molecule that is closely related to Rab9 in the molecular phylogenetic tree, causes deficiency in autophagosome and lysosome fusion in various cell types, including neuronal cells [46,47] and kidney epithelial cells [48]. However, its knockout only renders neuronal cells unresponsive to starvation for specific amino acids without defective lysosome function [46,47]. Herein, we characterize Rab9 as specifically antagonizing phenotypes that generate oligodendroglial cell-like widespread membranes in an oligodendroglial cell line. This conclusion is supported by the results demonstrating that the knockdown of Rab9 increases phenotypes with widespread membranes, along with an increased expression of oligodendroglial cell differentiation and myelination marker proteins.

Rab9A is a general Rab9 molecule, and Rab9B appears likely to perform a similar function as that of Rab9A [23,24,25,26,27,28]. However, Rab9A and Rab9B exhibit very different transcriptional profiles in brain cell types (see the Human Protein Atlas website, https://www.proteinatlas.org on 1 July 2024). While Rab9A is abundantly present in oligodendroglial lineage cells, Rab9B does not exhibit a specific profile across brain cell types. This observation underscores the close relationship between Rab9A and the differentiation of oligodendroglial lineage cells. Additionally, it is of note that a patient with mild HLD1 has a deficiency in a genomic region encompassing the entire PLP1 and Rab9B genes [49]. Since the genes encoding PLP1 and Rab9B are arranged in an antiparallel manner in the human and mouse genomes, patients with HLD1 caused by PLP1 deletion may also be affected by Rab9B deletion, potentially resulting in hypomyelination. Further studies are needed to clarify the association of Rab9 subfamily molecules with insufficient oligodendroglial cell differentiation and the hypomyelination phenotypes observed in HLD1.

The mechanism by which Rab9 decreases signaling related to ER stress remains unclear. In *Saccharomyces cerevisiae*, a Rab protein called Ypt1 directly controls ER stress and, in turn, UPR [47,48]. This protein regulates the stability of RNA encoding basic leucine zipper transcription factor HAC1 protein, which recognizes and binds to the UPR element in the promoter of UPR-regulated genes [47,48]. ER stress triggers the rapid localization of the Ypt1 protein to the cytoplasm and leads to its dissociation from pre-HAC1 mRNA. This change results in decreased pre-HAC1 mRNA degradation and, consequently, the activation of the UPR [50,51]. Although it is unlikely that the Ypt1 protein is a very close orthologue of the mammalian Rab9 protein, it is conceivable that Rab proteins, including Rab9, are related to ER stress and the UPR through the transport system around the ER in mammalian cells. Alternatively, mammalian cells, including neuronal cells, may respond to ER stress by modulating the expression levels of Rab proteins affecting organelle transport and membrane contacts around the ER. In fact, in inflammatory demyelinating diseases such as multiple sclerosis and experimental models, the upregulation of Rab32 affects the membranous contacts between the ER and mitochondria in response to ER stress [52]. In either mechanistic case, rather than what was previously expected, Rab proteins can be directly or indirectly responsible for ER stress and UPR.

Rab9 affects signaling pathways that reduce ER stress. One of the major pathways involves signalosome molecules around Akt [53,54,55]. Akt and phosphatidylinositol-3 kinase (PI3K) antagonistically regulate ER stress signaling. Intracellular signals dependent on Akt and PI3K can even inhibit ER stress-induced apoptosis [53,54,55]. However, it is unlikely that Rab9 inhibits Akt kinase and PI3K [53,54,55]. Another alternative signaling pathway involves the mitogen-activated protein kinase (MAPK) superfamily molecules [56,57,58]. Decreased Rab9 expression and/or activity may be involved in activating extracellular signal-regulated kinases (ERK) of the MAPK family. Rab9 may have an effect on the activity of MAPK/ERK, possibly reducing ER stress signaling. Among MAPK family molecules, there are stress-activated protein kinases (SAPKs)/c-Jun N-terminal kinases (JNKs) [56,57]. These subfamily molecules act directly downstream of ER stress-triggered inositol-requiring enzyme type 1 (IRE1) [29,30,31,32]. Thus, decreased Rab9 expression and/or activity may inactivate JNKs through IRE1.

We previously reported that Rab7B (also called Rab42) behaves antagonistically to ER stress signaling under the same experimental conditions as those used for Rab9 [39]. The knockdown of Rab7B recovers ER stress-induced morphological changes in oligodendroglial cells [39]. Since Rab7B is localized in organelles around the trans-Golgi network and the lysosome, the underlying molecular mechanisms governing the morphological changes in Rab9 and Rab7B may be similar in oligodendroglial cells. In the present study, we describe for the first time that Rab9 negatively regulates morphological changes. Notably, the knockdown of Rab9 helps cells undergo morphological changes. Further studies can promote our understanding not only of the detailed molecular mechanism by which Rab9 affects morphological changes but also of possible common mechanism(s) of Rab9 and Rab7B in these changes. Additional research is needed to understand how the knockdown of Rab9, as well as that of Rab7B, decreases ER stress signaling in primary oligodendroglial cells and in mice. Such studies may lead to the development of therapeutic target-specific medicines for hypomyelinating disease exhibiting incomplete or deficient differentiation.

## Figures and Tables

**Table 1:** Key materials.

Reagents or Sources	Company or Source	Cat. No.	Lot. No.	Concentration Used
Antibodies				
Anti-proteolipid protein 1 (PLP1)	Atlas Antibodies (Stockholm, Sweden)	HPA004128	8115828	Immunoblotting (IB), 1:500
Anti-myelin basic protein (MBP)	BioLegend (San Diego, CA, USA)	836504	B225469	IB, 1:500
Anti-GSTpi	MBL (Tokyo, Japan)	312	67	IB, 1:500
Anti-actin (also called pan-beta type actin)	MBL	M177-3	007	IB, 1:5000
Anti-eukaryotic initiation factor 2 alpha (eIF2a)	Santa Cruz Biotechnology (San Diego, CA, USA)	sc-13312	J1922	IB, 1:1000
Anti-phosphorylated eukaryotic initiation factor 2 alpha kinase (peIF2a)	PGI Proteintech Group Inc. (Rosemont, IL, USA)	28740-1-AP	00089246	IB, 1:1000
Anti-C/EBP homologous protein (CHOP)	PGI Proteintech Group, Inc.	15204-AP	00117318	IB, 1:1000
Anti-heat shock protein family A member 5 (HSPA5)	PGI Proteintech Group, Inc.	11587-AP	00085813	IB, 1:1000
Anti-phosphorylated Akt kinase (pS473)	CST (Danvers, MA, USA)	4060S	27	IB, 1:1000
Anti-Akt kinase	CST	4691T	28	IB, 1:1000
Anti-IgG (H+L chain) (Mouse) pAb-HRP	MBL	330	365	IB, 1:5000
Anti-IgG (H+L chain) (Rabbit) pAb-HRP	MBL	458	353	IB, 1:5000
Key chemicals				
Tunicamycin (TMC)	Cayman chemical campany (Arbor, MI, USA)	11445	0637439-4	100 ng/mL for typical experiments
Dimethyl sulfoxide (DMSO)	FUJIFILM Wako Pure Chemical Corporation (Tokyo, Japan)	047-29353	CDN0170	Less than 0.1%
Dithiothreitol (DTT)	Nacalai Tesque (Kyoto, Japan)	14128-46	not described	1 mM for typical experiments
Key reagents				
ScreenFect TM siRNA Transfection Reagent	FUJIFILM Wako Pure Chemical Corporation	292-75013	CAM0357	According to manufacturer’s instructions
ScreenFect TM Dilution Buffer	FUJIFILM Wako Pure Chemical Corporation	194-18181	SKF5794	According to manufacturer’s instructions
ImmunoStar TM Zeta	FUJIFILM Wako Pure Chemical Corporation	295-72404	WTL5319	According to manufacturer’s instructions
Chemi-Lumi TM One Ultra	Nacalai Tesque	11644-40	L2P1314	According to manufacturer’s instructions
Skim milk	FUJIFILM Wako Pure Chemical Corporation	190-12865	SKG4901	According to manufacturer’s instructions
Western blotting stripping solution	Nacalai Tesque	05364-55	L5M5218	According to manufacturer’s instructions
Fujifilm TM Sample buffer	FUJIFILM Wako Pure Chemical Corporation	191-13282	WDP4995	According to manufacturer’s instructions
Isogen	Nippon Gene (Tokyo, Japan)	311-02501	75009K	According to manufacturer’s instructions
5×PrimeScript master mix	TaKaRa Bio (Shiga, Japan)	RR036A	AIE0440A	According to manufacturer’s instructions
Gflex DNA polymerase	TaKaRa Bio	R060A	AL80564A	According to manufacturer’s instructions
2×Gflex PCR buffer (Mg^2+^, dNTP plus; with or without dye)	TaKaRa Bio	R060A	AL80564A	According to manufacturer’s instructions
TaKaRa Bio TM loading buffer	TaKaRa Bio	9157	A7201A	According to manufacturer’s instructions
Pre-stained Protein Markers (Broad Range) for SDS-PAGE	Nacalai Tesque	02525-35	L9M9989	According to manufacturer’s instructions
ExcelBand All Blue Regular Range Protein Marker	Cosmo Bio (Toyko, Japan)	PM1500	PM1500211500-5	According to manufacturer’s instructions
ExcelBrand 3 Color Regular Range Protein Marker	Smo Bio (New Taipei City, Taipei)	PM2500-2	PM25002112601-2	According to manufacturer’s instructions
Cells used				
FBD-102b cells (mouse oligodendrocyte progenitor cells)	Dr. Yasuhiro Tomo-oka (Riken, Saitama, Japan and Tokyo University of Science, Chiba, Japan)	N/A	N/A	1,000,000 cells per 6 cm dish culture
siRNA sequences (5′ to 3′)				
Sense chain for siLuciferase-105th (control siRNA) GCCAUUCUAUCCUCUAGAG-dTdT Antisense chain for siLuciferase-105th CUCUAGAGGAUAGAAUGGC-dTdT	This manuscript	N/A	N/A	50 nM per one transfection
Sense chain for siRab9-142th GAUCUGGAGGUGGACGGAC-dTdT Antisense chain for siRab9b-142th GUCCGUCCACCUCCAGAUC-dTdT	This manuscript	N/A	N/A	50 nM per one transfection
Sense chain for siRab9-199th GAACGCUUCCGAAGCCUGA-dTdT Antisense chain for siRab9b-199th UCAGGCUUCGGAAGCGUUC-dTdT	This manuscript	N/A	N/A	50 nM per one transfection
Sense chain for siRab9-212th GCCUGAGGACGCCAUUUUA-dTdT Antisense chain for siRab9b-212th UAAAAUGGCGUCCUCAGGC-dTdT	This manuscript	N/A	N/A	50 nM per one transfection
PCR primers (5′ to 3′)				
Sense primer for actin (internal control) ATGGATGACGATATCGCTGCGCTGGTC Antisense primer for actin CTAGAAGCACTTGCGGTGCACGATGGAG	This manuscript	N/A	N/A	250 nM per one reaction
Sense primer for Rab9 ATGGCAGGAAAATCGTCTCTTTTTAAAATAATTCTTCTTG Antisense primer for Rab9b TCAACAGCAAGATGAGTTTGGCTTGG	This manuscript	N/A	N/A	250 nM per one reaction
Plasmids				
pcDNA3.1(+)-c-eGFP-human PLP1/wild type PLP1	GenScript Japan (Tokyo, Japan)	TK797353	J908Z095G0-2	1 microgram per one transfection for 6 cm diameter-culture dish
pcDNA3.1(+)-c-eGFP-human PLP1 (A243V)/PLP1 p.Ala243Val	Genscript	TM203178	J357RPHAG0-1	1 microgram per one transfection for 6 cm diameter-culture dish

## Data Availability

The datasets used for the current study are available from the corresponding author upon reasonable request.

## Supplementary Materials

The following supporting information can be downloaded at: https://www.mdpi.com/article/10.3390/pathophysiology31030032/s1, Figure S1: Knockdown of Rab9 using the respective specific siRNAs; Figure S2: Tunicamycin stimulates ER stress signaling in FBD-102b cells; Figure S3: DTT stimulates ER stress signaling in FBD-102b cells; Figure S4: PLP1 with the A243V mutation decreases cell morphological changes; Figure S5: Knockdown of Rab9 ameliorates phenotypes in cells harboring PLP1 with the A243V mutation; Figure S6: Knockdown of Rab9 ameliorates the levels of Akt phosphorylation in ER stress-induced conditions; Figure S7: Original size blots in figures; Figure S8: Original size blots in supplemental figures; Table S1: Statistical data for graphs.
