# Supplementary material for: Knockdown of Rab9 Recovers Defective Morphological Differentiation Induced by Chemical ER Stress Inducer or PMD-Associated PLP1 Mutant Protein in FBD-102b Cells
Source: Pathophysiology. 2024 Aug 26;31(3):420–35. doi: 10.3390/pathophysiology31030032 (PMC11417737; doi:10.3390/pathophysiology31030032)
Supplement: Supplementary file 1 [file pathophysiology-31-00032-s001.zip › Supplemental figure legends.pdf]

Supplemental figure legends

Figure S1. Knockdown of Rab9 using the respective specific siRNAs. (A) FBD-102b cells were transfected with the respective siRNAs (142nd, 199th, and 212nd target sequences from A^1TG). Total RNA from these cells was subjected to RT-PCR to assess the knockdown efficacies. The siRNA targeting sequence from the 142nd was utilized for the following experiments.

Figure S2. Tunicamycin stimulates ER stress signaling in FBD-102b cells. (A, B) Following the induction of differentiation in the presence of 100 ng/ml of tunicamycin, FBD-102b cell lysates were immunoblotted with the respective antibodies against MBP and actin and statistically depicted as a percentage comparison (* $p < 0.05$; n = 3 blots). (C, D) Following the induction of differentiation in the presence of tunicamycin, cell lysates were immunoblotted with the respective antibodies against HSPA5 and actin and statistically depicted as a percentage comparison (* $p < 0.05$; n = 3 blots). (E, F) Following the induction of differentiation in the presence of tunicamycin, cell lysates were immunoblotted with the respective antibodies against CHOP and actin and statistically depicted as a percentage comparison (* $p < 0.05$; n = 3 blots). (G, H) Following the induction of differentiation in the presence of tunicamycin, cell lysates were immunoblotted with the respective antibodies against phosphorylated eIF2a (peIF2a), eIF2a, and actin and statistically depicted as a percentage comparison (* $p < 0.05$; n = 3 blots).

Figure S3. DTT stimulates ER stress signaling in FBD-102b cells. (A, B) Following the induction of differentiation in the presence of 1 mM DTT, FBD-102b cell lysates were immunoblotted with the respective antibodies against MBP and actin and statistically depicted as a percentage comparison (* $p < 0.05$; n = 3 blots). (C, D) Following the induction of differentiation in the presence of DTT, cell lysates were immunoblotted with the respective antibodies against HSPA5 and actin and statistically depicted as a percentage comparison (* $p < 0.05$; n = 3 blots). (E, F) Following the induction of differentiation in the presence of DTT, cell lysates were immunoblotted with the respective antibodies against CHOP and actin and statistically depicted as a percentage comparison (* $p < 0.05$; n = 3 blots). (G, H) Following the induction of differentiation in the presence of DTT, cell lysates were immunoblotted with the respective antibodies against phosphorylated eIF2a (peIF2a), eIF2a, and actin and statistically depicted as a percentage comparison (* $p < 0.05$; n = 3 blots).

Figure S4. PLP1 with the A243V mutation decreases cell morphological changes. (A) FBD-102b cells were transfected with the plasmids encoding wild type PLP1 or PLP1 with the A243V mutation. Following the induction of differentiation, cell morphologies were photographed and cells with differentiated oligodendroglial cell-like widespread membranes were statistically depicted at day 0 or 3 (** $p < 0.01$; n = 10 fields). Typical cell morphologies with differentiated oligodendroglial cell-like widespread membranes were surrounded by white dotted lines. (B) Cell lysates at day 3 following the induction of differentiation were immunoblotted with the respective antibodies against GSTpai,

HSPA5, and actin and statistically depicted as a percentage comparison (* $p < 0.01$; n = 3 blots).

Figure S5. Knockdown of Rab9 ameliorates phenotypes in cells harboring PLP1 with the A243V mutation. (A) FBD-102b cells were transfected with the plasmids encoding PLP1 with the A243V mutation plus luciferase siRNA (siLuc) or Rab9 siRNA (siRab9). Following the induction of differentiation, cell morphologies were photographed and cells with differentiated oligodendroglial cell-like widespread membranes were statistically depicted at day 0 or 3 (** $p < 0.01$; n = 10 fields). Typical cell morphologies with differentiated oligodendroglial cell-like widespread membranes were surrounded by white dotted lines. (B) Cell lysates at day 3 following the induction of differentiation were immunoblotted with the respective antibodies against GSTpi, HSPA5, and actin and statistically depicted as a percentage comparison (* $p < 0.01$; n = 3 blots).

Figure S6. Knockdown of Rab9 ameliorates the levels of Akt phosphorylation in ER stress-induced conditions. (A) FBD-102b cells were allowed to differentiate in the presence or absence (vehicle) of tunicamycin. Cell lysates were immunoblotted with the respective antibodies against phosphorylated Akt kinase (pAkt), Akt kinase (Akt), and actin and statistically depicted as a percentage comparison (* $p < 0.01$; n = 3 blots). (B) Cells were transfected with luciferase siRNA (siLuc, -) or Rab9 siRNA (siRab9) and allowed to differentiate in the presence (+) or absence (vehicle, -) of tunicamycin. Cell

lysates were immunoblotted with the respective antibodies against phosphorylated Akt kinase (pAkt), Akt kinase (Akt), and actin and statistically depicted as a percentage comparison (* $p < 0.01$; n = 3 blots). (C) Cells were allowed to differentiate in the presence or absence (vehicle) of DTT. Cell lysates were immunoblotted with the respective antibodies against phosphorylated Akt kinase (pAkt), Akt kinase (Akt), and actin and statistically depicted as a percentage comparison (* $p < 0.01$; n = 3 blots). (D) Cells were transfected with luciferase siRNA (siLuc, -) or Rab9 siRNA (siRab9) and allowed to differentiate in the presence (+) or absence (vehicle, -) of DTT. Cell lysates were immunoblotted with the respective antibodies against phosphorylated Akt kinase (pAkt), Akt kinase (Akt), and actin and statistically depicted as a percentage comparison (* $p < 0.01$; n = 3 blots). (E) Cells were transfected with the plasmids encoding wild type PLP1 or PLP1 with the A243V mutation and allowed to differentiate. Cell lysates were immunoblotted with the respective antibodies against phosphorylated Akt kinase (pAkt), Akt kinase (Akt), and actin and statistically depicted as a percentage comparison (* $p < 0.01$; n = 3 blots). (F) Following the induction of differentiation, cells were transfected with the plasmids encoding PLP1 with the A243V mutation plus luciferase siRNA (siLuc, -) or Rab9 siRNA (siRab9). Cell lysates were immunoblotted with the respective antibodies against phosphorylated Akt kinase (pAkt), Akt kinase (Akt), and actin and statistically depicted as a percentage comparison (* $p < 0.01$; n = 3 blots).

Figures S7. Original size blots in figures.

Figures S8 and S8's continued images. Original size blots in supplemental figures.

Table S1. Statistical data for graphs.
