# Supplementary figures and images for: Knockdown of Rab9 Recovers Defective Morphological Differentiation Induced by Chemical ER Stress Inducer or PMD-Associated PLP1 Mutant Protein in FBD-102b Cells
Source: Pathophysiology. 2024 Aug 26;31(3):420–35. doi: 10.3390/pathophysiology31030032 (PMC11417737; doi:10.3390/pathophysiology31030032)

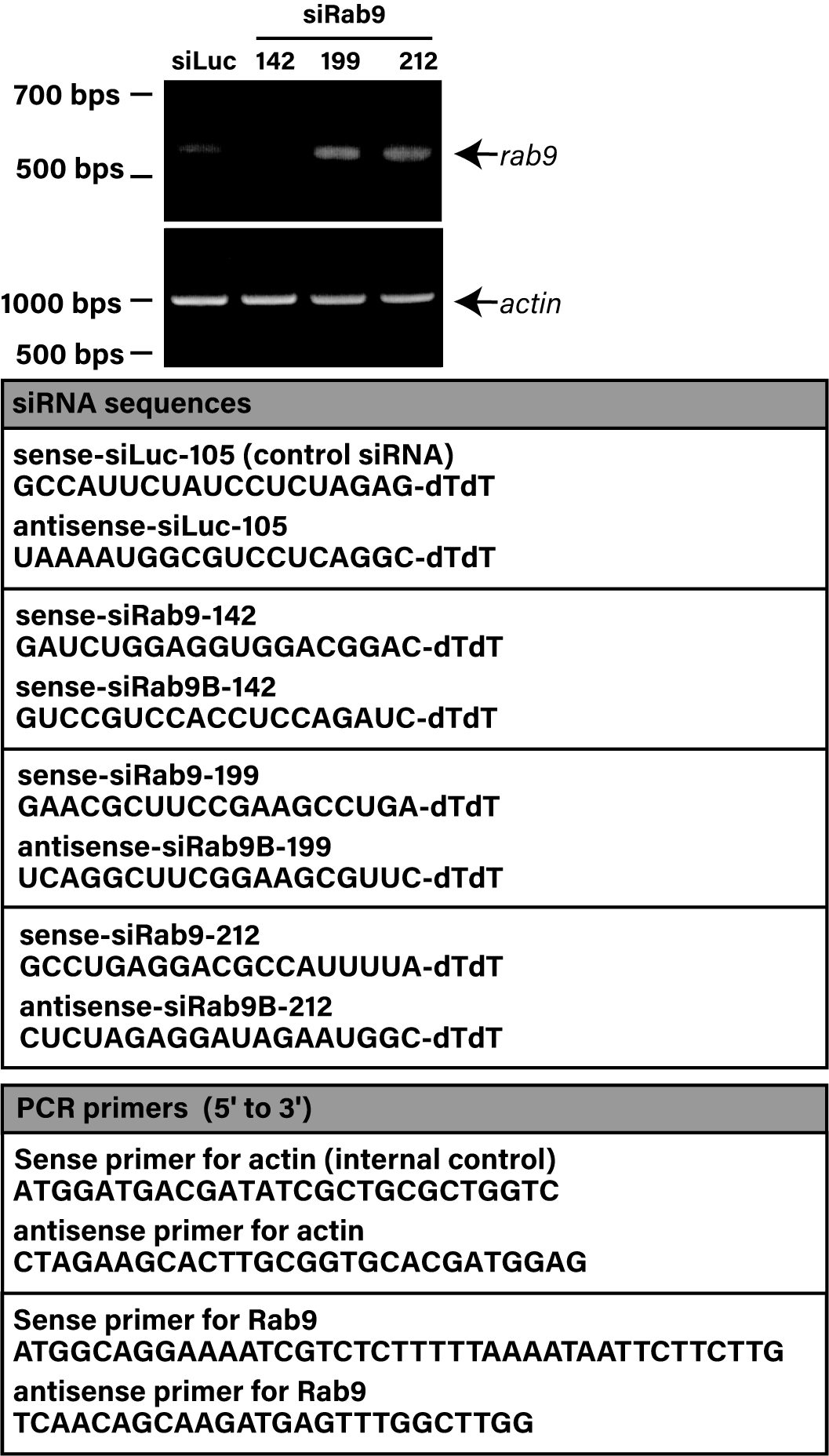

Supplement: Supplementary file 1 [file pathophysiology-31-00032-s001.zip › Figure S1.tif]

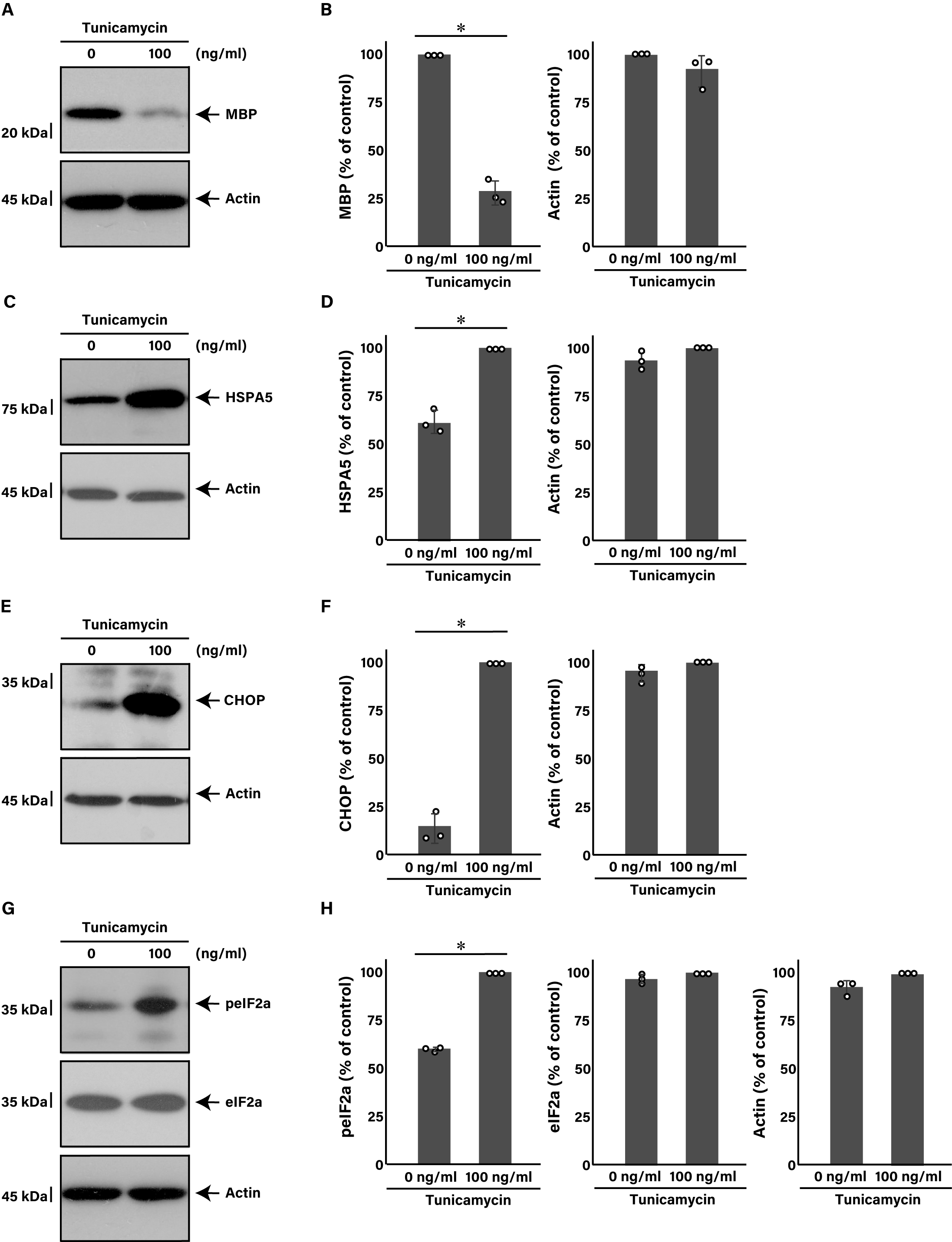

Supplement: Supplementary file 1 [file pathophysiology-31-00032-s001.zip › Figure S2.tif]

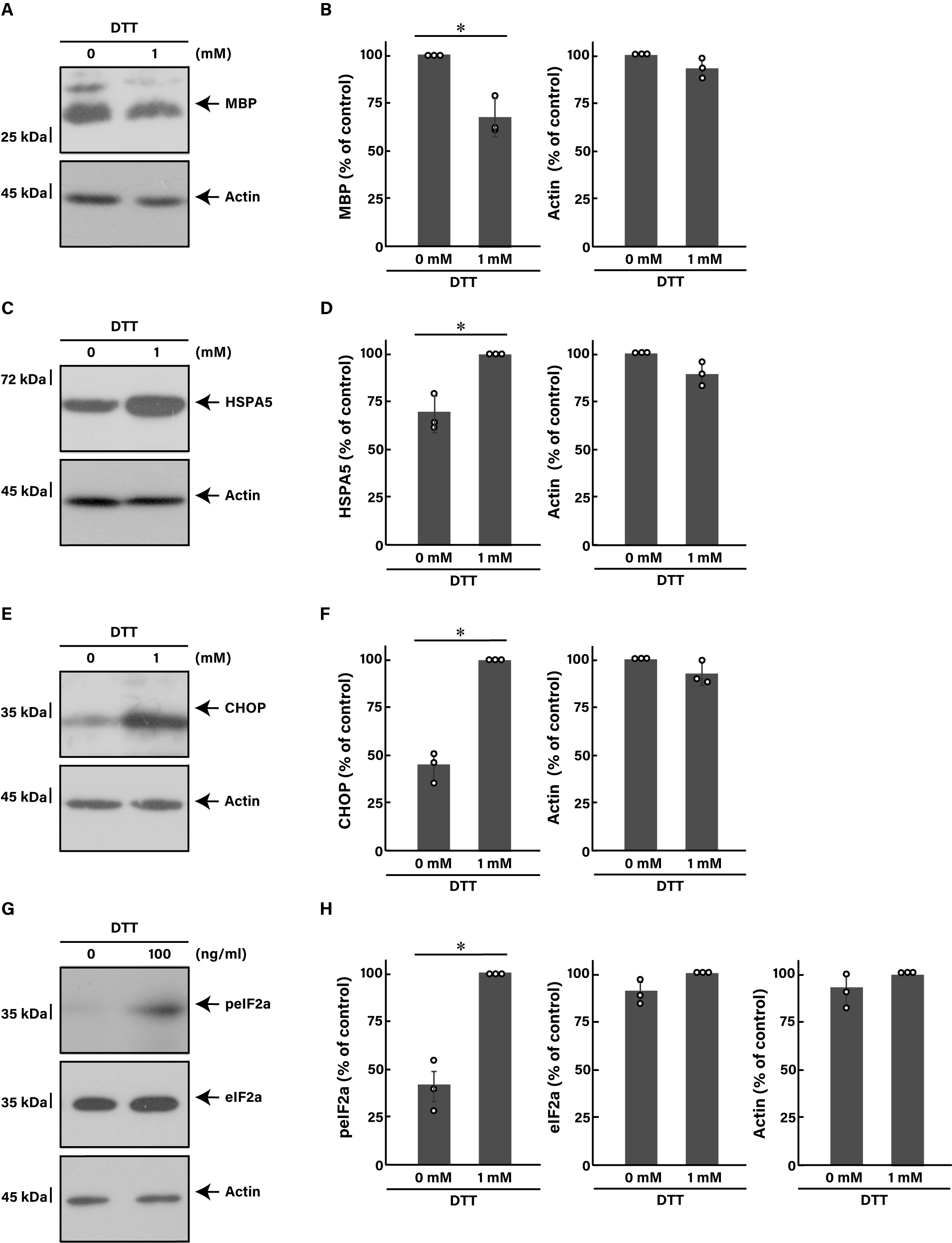

Supplement: Supplementary file 1 [file pathophysiology-31-00032-s001.zip › Figure S3.tif]

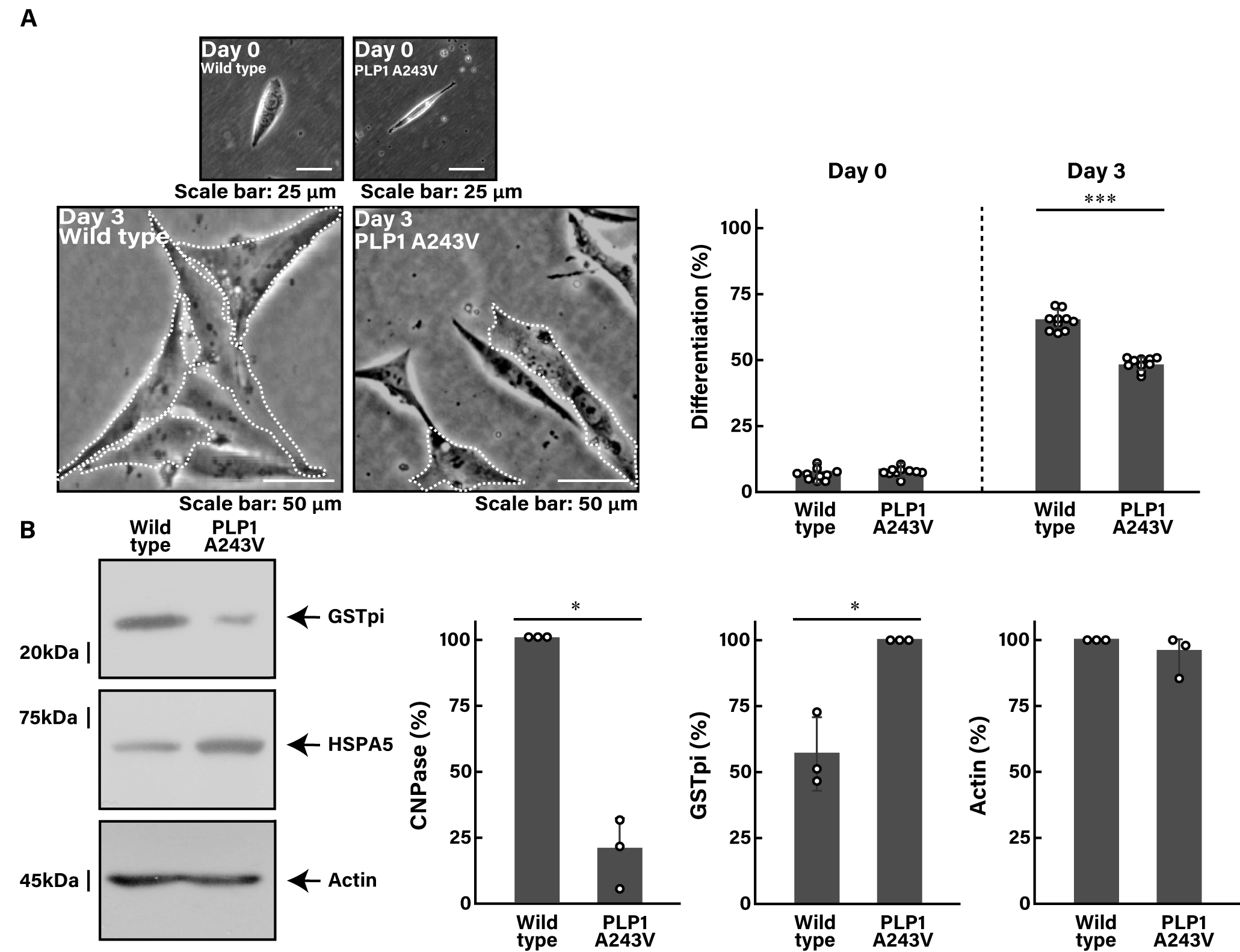

Supplement: Supplementary file 1 [file pathophysiology-31-00032-s001.zip › Figure S4.tif]

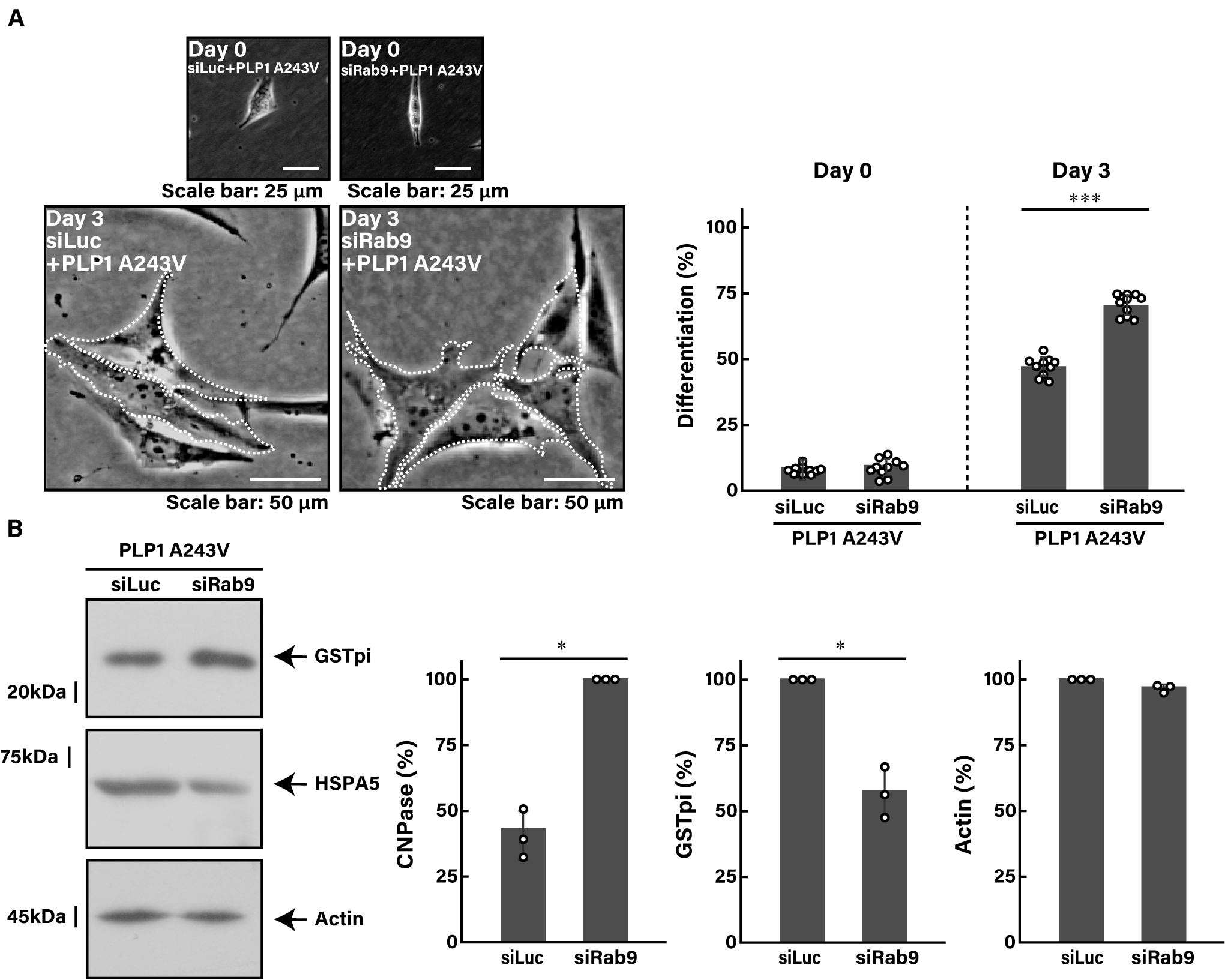

Supplement: Supplementary file 1 [file pathophysiology-31-00032-s001.zip › Figure S5.tif]

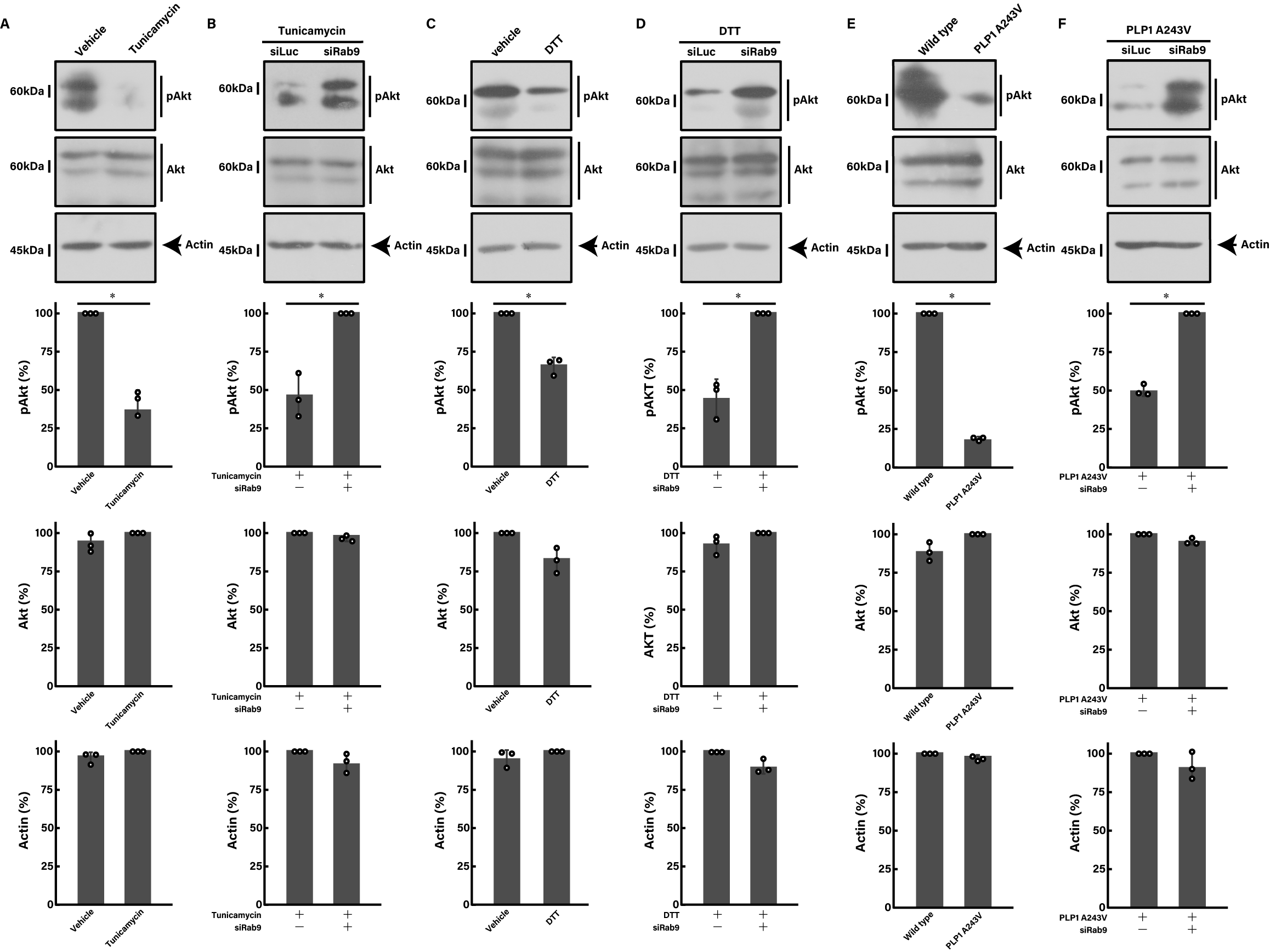

Supplement: Supplementary file 1 [file pathophysiology-31-00032-s001.zip › Figure S6.tif]

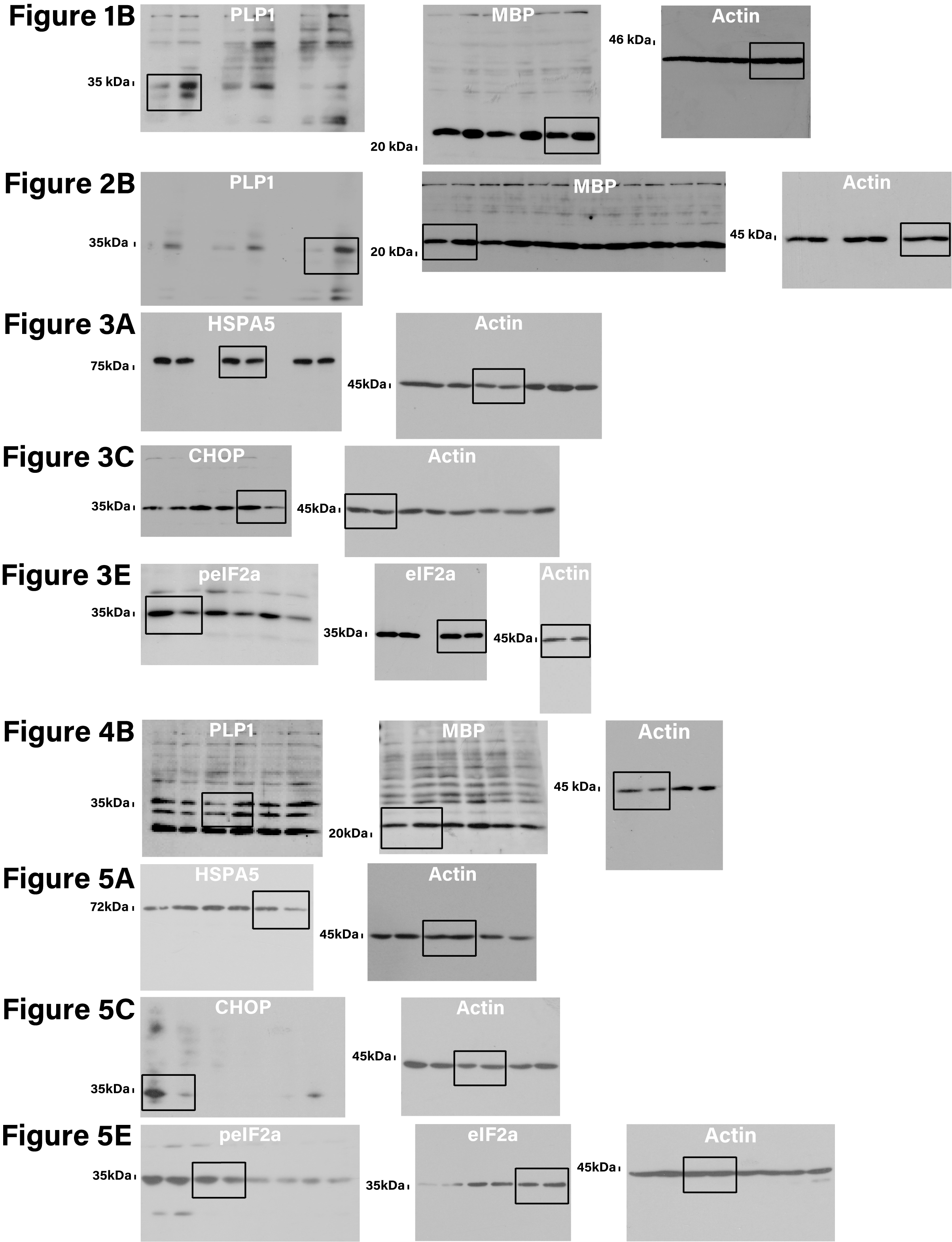

Supplement: Supplementary file 1 [file pathophysiology-31-00032-s001.zip › Figure S7.tif]

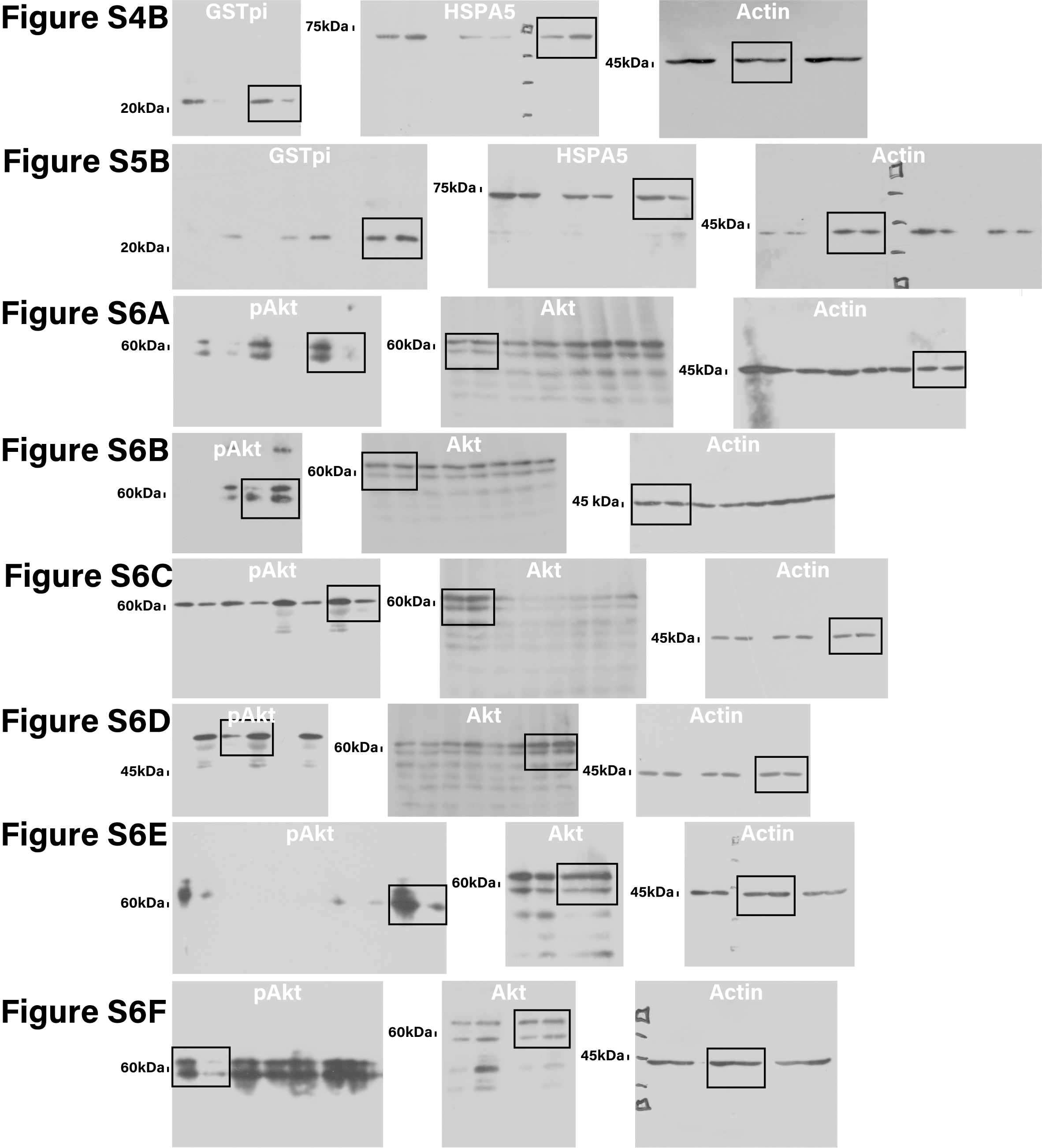

Supplement: Supplementary file 1 [file pathophysiology-31-00032-s001.zip › Figure S8's continued materials.tif]

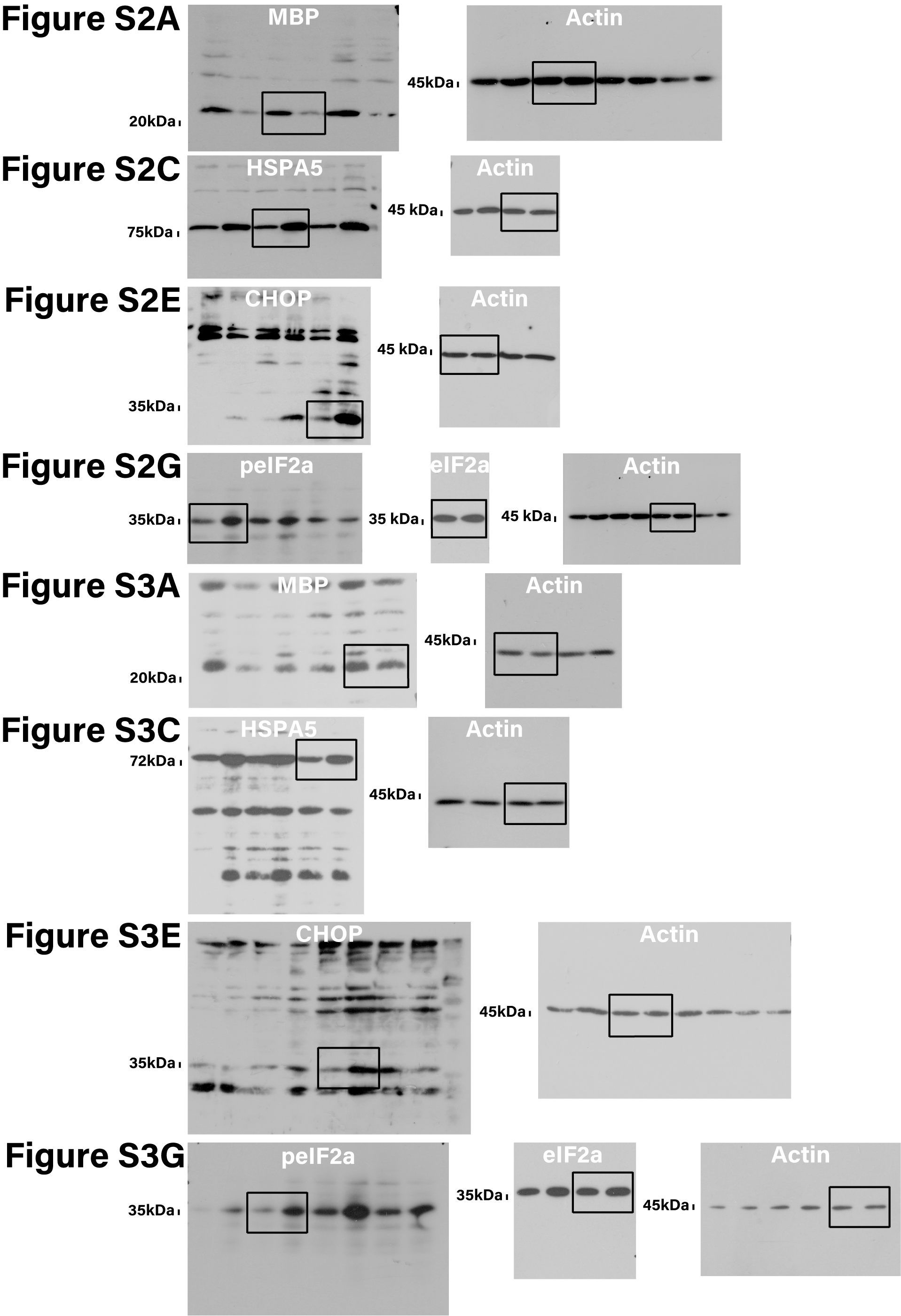

Supplement: Supplementary file 1 [file pathophysiology-31-00032-s001.zip › Figure S8.tif]
